# Bacteriuria and phenotypic antimicrobial susceptibility testing in 45 min by point-of-care Sysmex PA-100 System: first clinical evaluation

**DOI:** 10.1007/s10096-024-04862-3

**Published:** 2024-06-03

**Authors:** Carles Alonso-Tarrés, Carla Benjumea Moreno, Ferran Navarro, Aline C. Habison, Elisenda Gonzàlez-Bertran, Francisco Blanco, Jaume Borràs, Montserrat Garrigó, Jarob Saker

**Affiliations:** 1 Microbiology Laboratory, Fundació Puigvert, C/Cartagena 340-350, Barcelona, Spain; 2 Departament de Genètica i Microbiologia, Universitat Autònoma de Barcelona, Cerdanyola del Vallès, Barcelona, Spain; 3 Microbiology Laboratory, Hospital de la Santa Creu i Sant Pau, Barcelona, Spain; 4 New Business Development Department, Sysmex Europe SE, Norderstedt, Germany; 5 Primary Care Department, Sysmex Europe SE, Norderstedt, Germany; 6 Emergency Unit, Fundació Puigvert, Barcelona, Spain; 7 Medical Scientific Department, Sysmex Europe SE, Norderstedt, Germany

**Keywords:** Urinary tract infection (UTI), Antibiotic susceptibility test (AST), Phenotypic antibiotic susceptibility test (AST), Bacteriuria, Point-of-care, Nanofluidics, Antimicrobial resistance

## Abstract

**Purpose:**

This study compared the results of the new Sysmex PA-100 AST System, a point-of-care analyser, with routine microbiology for the detection of urinary tract infections (UTI) and performance of antimicrobial susceptibility tests (AST) directly from urine.

**Methods:**

Native urine samples from 278 female patients with suspected uncomplicated UTI were tested in the Sysmex PA-100 and with reference methods of routine microbiology: urine culture for bacteriuria and disc diffusion for AST.

**Results:**

The analyser delivered bacteriuria results in 15 min and AST results within 45 min. Sensitivity and specificity for detection of microbiologically confirmed bacteriuria were 84.0% (89/106; 95% CI: 75.6–90.4%) and 99.4% (155/156; 95% CI: 96.5–100%), respectively, for bacterial species within the analyser specifications. These are *Escherichia coli, Klebsiella pneumoniae, Proteus mirabilis, Enterococcus faecalis* and *Staphylococcus saprophyticus,* which are common species causing uncomplicated UTI. Overall categorical agreement (OCA) for AST results for the five antimicrobials tested in the Sysmex PA-100 (amoxicillin/clavulanic acid, ciprofloxacin, fosfomycin, nitrofurantoin and trimethoprim) ranged from 85.4% (70/82; 95%CI: 75.9–92.2%) for ciprofloxacin to 96.4% (81/84; 95% CI: 89.9–99.3%) for trimethoprim. The Sysmex PA-100 provided an optimal treatment recommendation in 218/278 cases (78.4%), against 162/278 (58.3%) of clinical decisions.

**Conclusion:**

This first clinical evaluation of the Sysmex PA-100 in a near-patient setting demonstrated that the analyser delivers phenotypic AST results within 45 min, which could enable rapid initiation of the correct targeted treatment with no further adjustment needed. The Sysmex PA-100 has the potential to significantly reduce ineffective or unnecessary antibiotic prescription in patients with UTI symptoms.

**Supplementary Information:**

The online version contains supplementary material available at 10.1007/s10096-024-04862-3.

## Introduction

The World Health Organisation (WHO) has raised concern regarding the overuse or use of inappropriate antimicrobials [1, 2]. In Europe 80–90% of antibiotics are dispensed in primary care settings [3] and in the United States at least 28% of the antibiotic courses prescribed each year are unnecessary [4]. This facilitates and escalates the spread of antimicrobial resistance (AMR), which is one of the greatest challenges to global public health and threatens our ability to treat common infections [1]. This prompted the World Health Assembly to adopt a multi-faceted global action plan with 5 main strategic objectives, including optimising the use of antimicrobial agents and investing in new diagnostic tools [5].

Urinary tract infections (UTIs) are among the most common bacterial infections with more than 400 million cases worldwide in 2019, imposing a significant burden on global health systems [6]. These patients usually present at a general practice or outpatient setting. The treating clinician does not have immediate knowledge of the causative organism since antimicrobial susceptibility tests (AST) in a clinical microbiology laboratory require 2–3 days, or in some instances, urine samples are not sent for laboratory analysis. Therefore, empirical treatment with a broad-spectrum antimicrobial is typically initiated, which may be ineffective. Once urine culture results are available, treatment is rarely adjusted since the patient may have recovered or is not present anymore. These sub-optimal empirical treatment situations create the opportunity for both treatment failure and/or an increase in drug-resistant uropathogens within the population [7, 8].

To optimise targeted antibiotic treatment for patients with UTI, the uropathogen must be identified and AST performed. Culture-based microbiological diagnostic methods are labour intensive, time-consuming and require trained personnel. These drawbacks have prompted the development of several laboratory and point-of-care (POC) approaches to supplement the culture-based methods [9, 10] and to optimize immediate treatment decisions. Genotypic AST methods detect the presence of specific resistance genes and produce results faster than conventional microbiology, but they are expensive and require knowledge of the target genes. Therefore, phenotypic AST methods remain the gold standard since the actual growth of the microorganism is monitored while in the presence of the antimicrobial agent [11]. Efforts have been made to decrease the time to obtain a definitive AST result, specifically by utilising microfluidic or nanofluidic technologies [12–15]. However, a gap still exists for accurate, cost-effective and reliable POC tests implementing this technology, which could rapidly detect the uropathogen and provide physicians with a susceptibility profile. The latter might then be used for immediate targeted treatment, avoiding the need for empirical antimicrobial use and de-escalation.

This study evaluated the diagnostic accuracy and clinical utility of the novel automated POC Sysmex PA-100 AST System (PA-100). It is designed for use in uncomplicated UTI. It detects bacteriuria in 15 min using fresh native urine and, in positive samples, performs phenotypic AST in an additional 15–30 min by automated phase-contrast monitoring of bacterial growth in the presence of an antimicrobial in Mueller–Hinton broth. This is the first time this technology has been assessed in a POC clinical setting for uncomplicated UTI.

## Methods

### Study design and patient population

This prospective, non-interventional study was conducted at Fundació Puigvert (FP) and Hospital de la Santa Creu i Sant Pau (HSP), Barcelona. Criteria for enrolment were female, non-pregnant patients, > 18 years old, presenting with current UTI symptoms in the urinary tract that had been present for < 7 days, with no antibiotic treatment within the previous 3 days, and who provided a clean-catch mid-stream urine sample. Patients with complicated UTI (e.g., anatomic abnormalities, renal disease, etc.) were excluded.

Informed consent was obtained from each patient. The study was conducted according to the Declaration of Helsinki and approved by the Ethics Committees of FP (13/07/2022) and HSP (07/09/2022). The study had no impact on the normal diagnostic and therapeutic procedures since clinicians were not informed of the PA-100 results during the study. Clinical information was obtained from personal interviews and electronic patient records.

### Sample size calculation

The expected sensitivity and specificity for detection of bacteriuria was 90% for both parameters. Assuming a prevalence of 0.6, significance level of 0.05, a precision of 0.05 and a drop-out of 10%, the calculated sample size for detection of bacteriuria was 385 patients.

The expected overall categorical agreement (OCA) for the AST was > 90% [16]. For a precision of 10%, the number of resistant strains to be collected was < 100 samples for all antimicrobials.

### Urine collection

Clean-catch midstream urine samples were collected and divided into 2 aliquots. In two cases samples were obtained from an in- and out-catheter. The first aliquot was processed within 30 min on the PA-100 (Sysmex Astrego, Uppsala, Sweden). The second aliquot was stored at 4 °C for < 4 h before reference method analysis.

### Test methods

#### PA-100

The fresh native urine sample was mixed and 400 µl pipetted into a PA-100 cartridge, which was inserted into the instrument.

The PA-100 automatically processes the sample after cartridge insertion. During the analysis, the urine sample flows through a filter into a nanofluidic chip. Bacterial cells, if present, are trapped in individual channels, whereas eukaryotic cells and other urine components, larger than bacteria, are filtered and do not enter the nanofluidic chip. After this loading step, Mueller Hinton broth II flows through reservoirs where dried antibiotics are kept. The growth medium, now containing a constant amount of antibiotics, flows into the channels where bacteria are trapped. All substances are prefilled in the cartridge. The incubation starts and the flow is kept constant throughout the analysis. The process is controlled by pressure regulation in the cartridge.

The nanofluidic chip contains approximately 11,000 nanochannels, grouped into 32 sections. Each section, containing a few hundred individual channels, is exposed to a different antibiotic and concentration of antibiotic. These antibiotic concentrations are used to calculate the resistance profile, according to a proprietary algorithm, which has been calibrated against the EUCAST Clinical Breakpoint tables [17].

During the analysis, the cartridge is moved by a mechanical system, which positions the nanofluidic chip in the field of view of a phase contrast microscope. Images are taken of all 32 sections in approximately 1-min intervals. A proprietary image analysis algorithm creates videos of the recorded images and calculates the growth rate of each of the sections. This growth rate is compared to the control section (no exposure to antibiotics).

The system detects the presence of bacteriuria (≥ 50,000 CFU/mL) and displays AST results as S (susceptible), I (susceptible, increased exposure), R (resistant) or not applicable, for amoxicillin/clavulanic acid, ciprofloxacin, fosfomycin, nitrofurantoin and trimethoprim, according to EUCAST [17]. A positive bacteriuria is stated without informing the user about the presumptive species identified by the analyzer and used to generate the AST result. The user receives a positive / negative notification only. Other results are possible: E (technical error) and LG (low growth: growth rate of the bacteria in the sample too low for accurate rapid AST results). The cartridge is designed for detection of the most frequent species causing uncomplicated UTI: *Escherichia coli, Klebsiella pneumoniae, Proteus mirabilis, Enterococcus faecalis* and *Staphylococcus saprophyticus.* An internal, non-disclosed proprietary algorithm is used to classify the bacteria into three different groups (Enterobacterales, *Staphylococcus* and *Enterococcus*) and to apply the relevant EUCAST breakpoints. Other non-fastidious bacteria might also be detected and displayed as positive. As the algorithm is not trained on those species, there is a chance that incorrect EUCAST breakpoints may be applied to bacteria uncommon in UTI potentially impacting those strains with resistance close to the breakpoint.

Furthermore, as *S. saprophyticus* and *Enterococcus* do not feature a breakpoint for oral fosfomycin, the analyzer will display “NA” for “not applicable” in the fosfomycin AST result if the algorithm concludes that the detected bacteria are members of the *Staphylococcus* or *Enterococcus* genera*.*

A more detailed description of the technology can be found elsewhere [14, 18–21].

#### Urinalysis

Urine cell counting was performed on an automated UN series Urinalysis flow cytometer analyser (Sysmex, Kobe, Japan).

#### Culture methods and analysis

Urine culture was initiated within 4 h of collection using 10 µL sterile disposable loops in Columbia Blood Agar (BA) (BD, Franklin Lakes, NJ, USA) and UTI urine chromogenic agar (ThermoFisher, Waltham, MA, USA) using the streaking technique. Also, 1 µL of urine was plated on an additional UTI chromogenic agar plate using the quantitative technique. Plates were incubated at 35 °C in air (UTI) or under 7% CO_2_ (BA) and read overnight and at 48 h.

The number of colonies on the quantitative agar plate was recorded as CFU/mL. Clinical-microbiological evaluation was made according to current standards [22]. Any clinically significant isolate was identified by VITEK2 (bioMérieux, Marcy-l’Étoile, France) or MALDI-TOF (Bruker, Billerica, MA, USA). Non-positive samples were classified as negative or contaminated by vaginal microbiota or another cause.

Samples were analysed for the presence of antimicrobials with a *Bacillus licheniformis* strain [23]. Any inhibition was recorded as probable presence of an antimicrobial in the sample.

#### Antimicrobial susceptibility tests

Susceptibility tests were performed by disc-diffusion on agar for all 5 antimicrobials [17] and interpreted according to EUCAST 2022 for uncomplicated UTI when available. Fosfomycin breakpoints for *Enterococci* are not provided by EUCAST and CLSI 2020 criteria were used instead [24]. For *Enterococci* trimethoprim susceptibility, a disc-diffusion ECOFF (Epidemiological cut-off value) diameter of 21 mm was used (EUCAST) and ampicillin susceptibility was used to test amoxicillin/clavulanic acid PA-100 results. MIC amoxicillin/clavulanic acid for *Enterococci* was done for any strain of this species resistant to this antimicrobial. Cefoxitin-deduced oxacillin susceptibility test results were used for amoxicillin/clavulanic acid in the *Staphylococci* disc-diffusion method (EUCAST)*.* In addition, broth microdilution was performed retrospectively on frozen isolates for amoxicillin/clavulanic acid, ciprofloxacin, nitrofurantoin and trimethoprim using Sensititre (ThermoFisher) and agar dilution for fosfomycin (Liofilchem, Roseto degli Abruzzi, Italy). Amoxicillin/clavulanic acid breakpoint for Enterobacterales was the 2022 EUCAST value for uncomplicated UTI (16 mm diameter for a 20–10 µg disc). Fosfomycin and nitrofurantoin disc-diffusion results for all Enterobacterales were interpreted according to the breakpoints given by EUCAST for *E. coli* uncomplicated UTI.

#### Detection of bacteriuria by PA-100 in clinical samples

Analytical performance of the PA-100 was calculated twice. One analysis was aligned to the specifications in the instructions for use (IFU) of the device (true positive defined as PA-100 positive and growth of *E. coli, K. pneumoniae, P. mirabilis, E. faecalis* or *S. saprophyticus* strain in culture; true negative defined as PA-100 negative and reference negative; detailed criteria in supplementary Table 1). The second analysis assessed the clinical performance of the PA-100 for all encountered species in samples considered positive by the microbiologist [22]. Samples with a positive *Bacillus licheniformis* inhibitor test would have been excluded, however, no sample showed inhibition [23].

#### Potential interference by host cells

Host cells were quantified using an UF series automated urine flow cytometer. A logistic regression was used to investigate the impact of the presence of host cells on a false negative bacteriuria result. The analysis focused on false negative results as this study only identified one false positive bacteriuria result by the PA-100.

#### Comparison of PA-100 AST results with routine microbiology on clinical samples

PA-100 AST results were compared with disc-diffusion. For discrepancies, broth microdilution was used as adjudicator for amoxicillin/clavulanic acid, ciprofloxacin, nitrofurantoin, trimethoprim, and agar dilution for fosfomycin. Results were analysed twice, once for samples containing of *E. coli, K. pneumoniae, P. mirabilis, S. saprophyticus* or *E. faecalis* and once for all samples (detailed criteria in supplementary Table 1). PA-100 AST results with an NA, LG or E message were excluded. Analysis was done according to ISO20776-2:2021 [25], in addition, OCA was reported.

#### Comparison with current clinical routine treatment

To assess the potential clinical usefulness of the PA-100, the treatment decisions made for enrolled patients were recorded. If the clinician prescribed an antibiotic other than nitrofurantoin, fosfomycin, amoxicillin/clavulanic acid, cotrimoxazole, trimethoprim or ciprofloxacin, the results of routine microbiology were utilized to assess potential resistance.

The priority list of antimicrobials for the hypothetical outcome was based on the assumption that the most frequent pathogen would be *E. coli* and that the analytical AST performance in a clinical setting was known. Antimicrobial side effects were also considered. If an antibiotic was reported as R, E or LG, the next antibiotic in the priority list was assessed. The order of antibiotics considered was nitrofurantoin > fosfomycin > trimethroprim > amoxicillin/clavulanic acid > ciprofloxacin.

#### Statistical analysis

Analysis was performed using MedCalc statistical analysis software version 20.104 (Medcalc Software, Ostend, Belgium). Comparison of proportions was done by N-1 chi-squared test. When comparing proportions, the confidence interval (CI) was calculated according to the recommended method [26]. CIs for sensitivity, specificity and accuracy were “exact” Clopper-Pearson CI. CIs for the likelihood ratios were calculated using the “Log method”.

## Results

### Study population

A total of 307 patients were enrolled. Twenty-nine patients with complicated UTI were excluded retrospectively. The final study population consisted of 278 patients, 199 enrolled at FP between September 2022 and March 2023, and 79 enrolled at HSP between September 2022 and January 2023. Patient characteristics are summarized in Table 1.

**Table 1:** Patient characteristics. Data are given for all patients recruited at Sant Pau and at Fundació Puigvert. Relative frequency of each parameter is in brackets

Patients	278
Age (median & 95% CI)	54 (45 – 60)
Cystitis	194 (69.8%)
Pyelonephritis	9 (3.2%)
Antibiotics prescribed	191 (68.7%)
Pretreatment with Antibiotics in last 7 days	8 (2.9%)
Use of painkillers	142 (51.1%)
Diabetes mellitus	17 (6.1%)
Dysuria	188 (67.6%)
Frequency	203 (73%)
Urgency	129 (46.4%)
Fever	16 (5.8%)
Flank pain	158 (56.8%)
Suprapubic pain	202 (72.7%)
Nausea/vomiting	64 (23%)
General discomfort	107 (38.5%)
History of recurrent UTI	97 (34.9%)
Previous urine culture at any time point	111 (39.9%)

### Detection of bacteriuria

The performance of the PA-100 was compared to the microbiological reference (Tables 2 and 3). Under IFU specifications bacteriuria detection by the PA-100 in positive urine cultures was 89 out of 106 (sensitivity 84.0%, CI:75.6% to 90.4%). Of 94 samples positive for Gram-negative bacteria 85 were detected, while 4 out of 8 Gram-positive bacteria were detected. Culture negative urines, confirmed as negative by the PA-100, were 155 out of 156 (specificity 99.4%, CI: 96.5% to 99.8%). Overall accuracy was 93.1% (89.1% to 95.9%).

**Table 2:** Summary of microbiological results. Species are sorted by Gram stain, frequency of isolation and name. Species in bold are covered under IFU claims. “All” describes the frequency of encountered strains irrespective of their concentration in the urine sample or their inclusion in the IFU. Frequency of isolates above 50,000 CFU/mL and detection rate of PA-100 are highlighted in separate columns

	Microbiological reference	All	IFU specification	Detected by PA-100
Gram negative	***Escherichia coli***	91	74	65
	***Klebsiella pneumoniae***	21	17	18
	***Proteus mirabilis***	4	3	2
	*Pseudomonas aeruginosa*	3	-	1
	*Citrobacter koseri*	1	-	0
	*Klebsiella oxytoca*	1	-	0
	*Providencia rettgeri*	1	-	1
Gram positive	***Staphylococcus saprophyticus***	7	7	2
	***Enterococcus faecalis***	4	1	2
	*Streptococcus anginosus*	3	-	0
	*Streptococcus agalactiae*	2	-	0
	*Aerococcus urinae*	1	-	1
	*Aerococcus viridans*	1	-	1
	*Lactococcus garvieae*	1	-	1
	*Staphylococcus warneri*	1	-	0
	*Candida glabrata*	1	-	0
	All Gram negatives	122	94	87
	All Gram positives	20	8	7
	Total	143	102	94

**Table 3:** Comparison of detection of bacteriuria by PA-100 compared to microbiological investigation. The detection of bacteriuria by the PA-100 was compared retrospectively with microbiological evaluation. For the analysis in a) only the five species (*E. coli, P. mirabilis, K. pneumoniae, S. saprophyticus, E. faecalis*) and quantification on Brilliance agar above 50,000 CFU/mL as specified in the instructions for use (IFU) were considered, b) shows performance regarding all bacterial species encountered in the study

According to IFU specifications		Microbiological reference		
		positive	negative	total
PA-100	positive	89	1	90 (34.4%)
	negative	17	155	172 (65.6%)
	total	106 (40.5%)	156 (59.5%)	262
Sensitivity (95% CI)		84.0% (75.6—90.4)		
Specificity (95% CI)		99.4% (96.5–100)		
Accuracy/OCA (95% CI)		93.1% (89.1–95.9)		
PPV (95% CI)		98.9% (92.7–99.8)		
NPV (95% CI)		90.1% (85.5–93.4)		
All positive samples, no minimum bact. count		Microbiological reference		
		positive	negative	total
PA-100	positive	94	1	95 (34.2%)
	negative	49	134	183 (65.8%)
	total	143 (51.4%)	135 (48.6%)	278
Sensitivity (95% CI)		65.7% (57.3–73.5)		
Specificity (95% CI)		99.3% (95.9–100)		
Accuracy/OCA (95% CI)		82.0% (77.0—86.3)		
PPV (95% CI)		99 .0% (39.0–99.9)		
NPV (95% CI)		73.2% (68.5–77.4)		

CI: Confidence interval. OCA: Overall categorical agreement. PPV: Positive predictive value. NPV: Negative predictive value

Considering all samples judged as positive by the microbiologist irrespective of species or count (Tables 2 and 3), the PA-100 sensitivity was 65.7% (94/143, CI: 57.3% to 73.5%). This lower sensitivity was associated with a positive culture at concentrations < 50,000 CFU/mL (21 cases) or a positive culture for species outside the analyser specifications (1 *Citrobacter koseri*, 1 *Klebsiella oxytoca*, 2 *Pseudomonas aeruginosa*, 3 *Streptococcus anginosus*, 2 *Streptococcus agalactiae* and 1 *Staphylococcus warneri*). Of 122 samples with Gram-negative bacteria, 87 were detected, while 7 out of 20 samples with Gram-positive bacteria were detected. Specificity was 99.3% (134 of 135, CI 95.9% to 100%) and accuracy 82.0% (77.0% to 86.3%).

### Potential interference by host cells

Fragments of host blood cells or epithelial cells may be present in urine samples, and these could enter or block the nanofluidic channels, preventing the entrapment of bacteria. When tested with a logistic regression model, red blood cell, white blood cell and epithelial cell counts in the urine were not associated with false negative results (p values were 0.1146, 0.7760 and 0.2303, respectively).

### Performance of PA-100 antimicrobial susceptibility test results

Performance of the PA-100 AST on fresh urine samples within IFU specifications is described in Table 4. Similar results were obtained for the performance of the PA-100 AST on all fresh urine samples from the study (Table 5). OCA over all antibiotics was 94.6% (CI 91.9%-96.6%) and 94.0% (CI 91.2%-96.1%), respectively.

**Table 4:** Performance of PA-100 antibiotic susceptibility test on urine samples within IFU specifications. The PA-100 results for AST were compared to combined disc diffusion and broth microdilution for amoxicillin/clavulanic acid (AMC), ciprofloxacin (CIP), nitrofurantoin (NIT) and trimethoprim (TRI), as well as disc diffusion and agar dilution for fosfomycin (FOS). S: susceptible, I: susceptible, increased exposure, R: resistant based on standard dosing regimen. Overall categorical agreement (OCA), as well as sensitivity and specificity for the detection of antibiotic resistance are reported with 95% confidence interval (CI) in brackets. PPV: Positive predictive value. NPV: Negative predictive value. Display of “NA” as an AST result refers to an antimicrobial that is not applicable, according to EUCAST, for the species identified by the PA-100

Microbiological reference	PA-100	AMC	CIP	FOS	NIT	TRI
S	S	54 (92%)	58 (92%)	50 (96%)	72 (96%)	55 (96%)
	I		4 (6%)			
	R	5 (8%)	1 (2%)	2 (4%)	3 (4%)	2 (4%)
	total	59	63	52	75	57
I	S		1 (33%)			
	I		0 (0%)			
	R		2 (67%)			
	total		3			
R	S	3 (17%)	1 (6%)	2 (13%)	1 (20%)	1 (4%)
	I		3 (19%)			
	R	15 (83%)	12 (75%)	13 (87%)	4 (80%)	26 (96%)
	total	18	16	15	5	27
Frequency of no result due to NA		5	0	6	0	1
OCA (95% CI)		89.6% (80.6–95.4)	85.4% (75.9–92.2)	94.0% (85.4–98.4)	95.0% (87.7–98.6)	96.4% (89.9–99.3)
Sensitivity (95% CI)		83.3% (58.6–96.4)	75.0% (47.6–92.7)	86.7% (59.5–98.3)	80.0% (28.4–99.5)	96.3% (81.0–99.9)
Specificity (95% CI)		91.5% (81.3–97.2)	92.1% (82.4–97.4)	96.2% (86.8–99.5)	96.0% (88.8–99.2)	96.5% (87.9–99.6)
PPV (95% CI)		75.0% (57.7–86.8)	80.0% (56.1–92.69	86.7% (62.2–96.3)	57.1% (28.8–81.5)	92.9% (76.9–98.1)
NPV (95% CI)		94.7% (86.5–98.1)	94.0% (87.1–97.4)	96.2% (87.3–98.9)	98.6% (92.6–99.8)	98.2% (88.9–99.7)

**Table 5:** Performance of PA-100 antibiotic susceptibility test on all fresh urine samples from the study. The PA-100 results for AST were compared to combined disc diffusion and broth microdilution for amoxicillin/clavulanic acid (AMC), ciprofloxacin (CIP), nitrofurantoin (NIT) and trimethoprim (TRI), as well as disc diffusion and agar dilution for Fosfomycin (FOS). S: susceptible, I: susceptible, increased exposure, R: resistant based on standard dosing regimen. Overall categorical agreement (OCA), as well as sensitivity and specificity for the detection of antibiotic resistance are reported with 95% confidence interval (CI) in brackets. PPV: Positive predictive value. NPV: Negative predictive value. Display of “NA” as an AST result refers to an antimicrobial that is not applicable, according to EUCAST, for the species identified by the PA-100.

Microbiological reference	PA-100	AMC	CIP	FOS	NIT	TRI
S	S	55 (92%)	60 (91%)	50 (96%)	74 (96%)	56 (97%)
	I		5 (8%)			
	R	5 (8%)	1 (2%)	2 (4%)	3 (4%)	2 (3%)
	total	60	66	52	77	58
I	S		1 (25%)			
	I		0 (0%)			
	R		3 (75%)			
	total		4			
R	S	4 (21%)	1 (6%)	2 (13%)	1 (17%)	3 (10%)
	I		3 (18%)			
	R	15 (79%)	12 (75%)	14 (88%)	5 (83%)	27 (90%)
	total	19	16	16	6	30
Frequency of no result due to NA		7	0	9	0	2
OCA (95% CI)		88.6% (79.5–94.7)	83.7% (74.2–90.8)	94.1% (85.6–98.4)	95.2% (88.1–98.7)	94.3% (87.2–98.1)
Sensitivity (95% CI)		79.0% (54.4–94.0)	75.0% (47.6–92.7)	87.5% (61.7–98.5)	83.3% (35.9–99.6)	90.0% (73.5–97.9)
Specificity (95% CI)		91.7% (81.6–97.2)	90.1% (81.3–96.6)	96.2% (86.8–99.5)	96.1% (89.0–99.2)	96.6% (88.1–99.6)
PPV (95% CI)		75.0% (55.7–87.8)	75.0% (52.6–89.0)	87.5% (64.0–96.5)	62.5% (34.2–84.2)	93.1% (77.5–98.2)
NPV (95% CI)		93.2% (85.2–97.1)	94.3% (87.6–97.5)	96.2% (87.3–98.9)	98.7% (92.5–99.8)	94.9% (86.4–98.2)

Performance characteristics of the AST are stated according to ISO 20776–2 2021. In addition, we investigated the frequency of minor errors (mE), major errors (ME) and very major errors (VME). For amoxicillin/clavulanic acid the frequency of ME was 6.5% (2.1 – 14.5) and of VME was 3.9% (0.8 – 11). For ciprofloxacin the frequency of mE was 12.2% (6 – 21.3), of ME was 3.7% (0.8 – 10.4) and of VME was 1.2% (0 – 6.6). For fosfomycin the frequency of ME was 3% (0.4 – 10.4) and of VME was 3% (0.4 – 10.4). For nitrofurantoin the frequency of ME was 3.8% (0.8 – 10.6) and of VME was 1.3% (0—6.8). For trimethoprim the frequency of ME was 2.4% (0.3 – 8.4) and of VME was 1.2% (0 – 6.5).

Microdilution results in agreement with PA-100 results changed 0 S and 11 R results for amoxicillin/clavulanic acid achieved by disc diffusion, 0 S and 1 R results for nitrofurantoin, 0 S and 0 R results for trimethoprim, 0 S, 1 R and 0 I results for ciprofloxacin, 0 S and 6 R results for fosfomycin.

Oral fosfomycin breakpoints are not provided by EUCAST for *S. saprophyticus* and *Enterococcus*. Out of three total *Enterococcus* infections two were below the cut-off of 50,000 CFU/mL. The sample with more than 50,000 CFU/mL and one of the samples with less were detected by the system and correctly flagged NA for fosfomycin. Out of 7 *S. saprophyticus* infections 7 were above the cut-off of 50,000 CFU/ml. Two of these samples were detected by the PA-100 as bacteriuria positive. One sample was correctly flagged as NA and one sample resulted in no AST result due to LG being displayed. In conclusion, no sample received an incorrect S or R result for fosfomycin.

In general, NA for amoxicillin/clavulanic acid was flagged in one case each of *Aerococcus urinae, Aerococcus viridan, Enterococcus faecalis, Escherichia coli, Klebsiella pneumoniae, Staphylococcus epidermis* and *Staphylococcus saphrophyticus.* NA for fosfomycin was flagged in one case each of *Aerococcus urinae, Aerococcus viridans* group*, Escherichia coli, Klebsiella pneumoniae, Lactococcus garvieae, Staphylococcus epidermidis* and *Staphylococcus saphrophyticus,* and two cases of *Enterococcus faecalis.* NA for trimethroprim was flagged in one case of *Enterococcus faecalis* and one case of *Lactococcus garvieae.*

The frequency of invalid results due to LG, despite a positive result for bacteriuria, or technical errors, was 8.9% for amoxicillin/clavulanic acid, 7.8% for ciprofloxacin, 19.1% for fosfomycin, 10.1% for nitrofurantoin and 4.5% for trimethoprim.

### Comparison of PA-100 results with current best practice

To provide context to the analytical performance of the PA-100, actionable recommendations of the analyser, as well as clinical treatment decisions, were compared with clinical microbiology results as a reference (Table 6). Clinicians did not have access to the PA-100 results but based their decisions on current clinical best practice. In reference bacteriuria-negative patients, PA-100 recommendation not to treat was significantly more frequent than actual clinical decisions (99.3% vs 49.6%; p < 0.0001). In reference bacteriuria-positive patients, PA-100 recommendation not to treat was also significantly more frequent than actual clinical decisions (34.3% vs 15.4%; p = 0.0002). Importantly, however, considering the microbiological resistance profile of the isolated bacteria, the PA-100 recommendation for an inappropriate antibiotic was significantly lower than actual clinical decisions (2.1% vs 18.2%; p < 0.0001).

**Table 6:** Comparison between clinical decision and PA-100 AST results. Performance of actual clinical decision making and PA-100 treatment recommendations when compared to microbiological investigation. Optimal treatment corresponds to a) negative urine culture, patients not treated and b) positive urine culture, patient treated with an antimicrobial to which the uropathogen was susceptible according to the final routine culture report. Suboptimal treatment includes a) negative urine culture, patient treated with antimicrobial and b) positive urine culture, patient not treated or treated with an antimicrobial to which the uropathogen was resistant.

Urine culture		Clinical decision	PA-100 recommendation	p value
Negative	No antimicrobial given / PA negative	67 (49.6%)	134 (99.3%)	** < 0.0001**
	Antimicrobial given / PA positive	68 (50.4%)	1 (0.7%)	** < 0.0001**
	**subtotal**	**135**	**135**	
Positive	Appropriate decision / recommendation (susceptible)	93 (65%)	84 (58.7%)	0.2736
	Appropriate decision / recommendation (increased exp.)	2 (1.4%)	0 (0%)	0.1564
	Inappropriate decision / recommendation (resistant)	26 (18.2%)	3 (2.1%)	** < 0.0001**
	Resistant against all antimicrobials on PA-100		2^a^ (1.4%)	
	No AST result on PA-100		5 (3.5%)	
	Decision / recommendation to not treat (suspected negative)	22 (15.4%)	49 (34.3%)	**0.0002**
	**subtotal**	**143**	**143**	
Optimal treatment		162 (58.3%)	218 (78.4%)	** < 0.0001**
Suboptimal treatment		116 (41.7%)	53 (19.1%)	** < 0.0001**
**total**		**278**	**278**	

^a^Clinical decision making cannot consider the appearance of multiple resistant strains

Overall frequency of optimal treatment defined as a) negative urine culture, patients not treated and b) positive urine culture, patients treated with an antimicrobial to which the pathogen was susceptible according to the final routine culture report, was significantly higher for the PA-100 recommendation compared to the clinical decision (78.4% vs 58.3%; p < 0.0001).

## Discussion

This study documents for the first time the performance of the PA-100 in a clinical setting using patient urine samples. Previous publications were limited to isolates [14, 18]. The PA-100 is not intended as a substitute for a microbiology laboratory, but as a diagnostic tool to improve the standard of care in near-patient settings. The instrument detects bacteriuria and performs phenotypic AST by automated phase-contrast microscopy monitoring. The system is easy to use, and interpretation of results does not require knowledge of clinical microbiology. The PA-100 uses fresh urine samples within 30 min of collection, which is commensurate with its deployment in a POC setting where rapid results are required. In addition, no pretreatment of urine is necessary.

The PA-100 detected bacteriuria and provided an AST profile under IFU specifications with 84.0% sensitivity and 99.4% specificity. For positive urine cultures outside IFU specifications sensitivity was lower (65.7%), but specificity remained high (99.3%). Challenges were encountered in detecting Gram-positive strains (5 out of 7 *S. saprophyticus,* 2 out of 4 *E. faecalis* and 3 out of 3 *S. anginosus* were missed). Furthermore, the PA-100 detects bacteriuria when samples exhibit > 50,000 CFU/mL, whereas the reference criteria set by the microbiology laboratory classified samples as positive at lower levels of bacteriuria, according to current clinical practice [27].

Overall categorical agreement for the five antimicrobials evaluated in urine samples under IFU specifications was satisfactory for its POC design (94.6%, CI: 91.9%-96.6%). While sensitivity of the AST results appeared to be low (ranging from 75% to 96.3%), the VITEK2 had a similar performance when compared to our microbiological reference (data not shown). This is in line with the performance of other automated AST systems [28]. In a clinical setting the PA-100 uses native urine samples, as opposed to isolates, making it more challenging to provide a correct result. It is important to highlight that the NPV for resistance in our cohort was > 93% for all antibiotics tested, which indicates that a susceptible test result could reliably be used for treatment decisions. Also, the very good NPV is consistent with its use in uncomplicated UTI, where AMR is typically low, and it would even improve in settings where AMR is not as frequent as in this study population.

As described in the methods, in samples where bacteria like *Pseudomonas, Aerococcus,* or *Lactococcus* were detected as positive by the system, an incorrect AST reference might be used by the PA-100. Furthermore, the PA-100 does not distinguish between different species of Enterobacterales and thus *E. coli* fosfomycin and nitrofurantoin breakpoints are always used, irrespective of the final microorganism identification. Since the system does not display which microorganism is assumed, there is also no leverage to immediately reflect on this. This problem of course only applies to strains with resistance features close to their respective breakpoints, but it is a potential drawback of the system.

The PA-100 markedly outperformed current clinical decisions. Overall, the frequency of optimally treated patients, i.e., non-infected patients that were not exposed to antimicrobials and infected patients that were treated with an effective antimicrobial, could have improved significantly from 58.3% to 78.4% (p < 0.0001) if the physician had had access to the PA-100 AST results during consultation. It is important to note that this comparison is hypothetical, and that the actual clinical impact in a healthcare setting still needs to be demonstrated.

Despite sensitivity not being high enough to reliably rule out infection (NPV of 73.2% in this study), the excellent specificity of the PA-100 and the rapid availability of accurate diagnostic and AST data would enable physicians attending to patients with UTI to implement correct targeted treatment in positive cases without delay, avoiding the overuse of broad-spectrum antimicrobials and the subsequent need to de-escalate. In this study 18.2% of the antimicrobials prescribed empirically in bacteriuria-positive patients were ineffective due to bacterial resistance, and utilising PA-100 AST guidance would have decreased this to 2.1% (p < 0.0001). This is especially important in uncomplicated UTI, where treatment adjustment is seldom done, making this disease an important source of resistant strains and exacerbating possible overtreatment by physicians (36.0%, 68/189 of all antimicrobials dispensed in this study were for culture-negative patients).

A limitation of the PA-100 was the relatively high frequency of samples with bacterial growth too low to perform an AST for one of the antimicrobials in the cartridge. However, switching to a different antimicrobial for which an AST was reported, was an obvious solution for this problem, and results not available for all antimicrobials only occurred in five positive PA-100 samples.

Follow-up interventional studies in which the antimicrobials are prioritised depending on the local prevalence of resistant bacteria will be useful to establish the actual clinical impact of the PA-100. It would also be informative to repeat this study in a setting with low prevalence of resistance and strict antibiotic prescription behaviour, as in such settings the PA-100 could potentially detect otherwise untreated bacteriuria.

In conclusion, the PA-100 has implications for clinical practice since it provides bacteriuria and phenotypic AST results within 45 min to guide physicians in quickly making informed decisions and thus significantly improve treatment of patients with UTI. It also has the potential to reduce the inappropriate use of antimicrobials and, hence, overall AMR frequency in the population.

### Supplementary Information

Below is the link to the electronic supplementary material.

Supplementary file1 (DOCX 13 KB)

## Data Availability

The datasets generated during and/or analysed during the current study are not publicly available due intellectual property but are available from the corresponding author on reasonable request.
