# Supplementary material for: Bacteriuria and phenotypic antimicrobial susceptibility testing in 45 min by point-of-care Sysmex PA-100 System: first clinical evaluation
Source: Eur J Clin Microbiol Infect Dis. 2024 Jun 3;43(8):1533–43. doi: 10.1007/s10096-024-04862-3 (PMC11271345; doi:10.1007/s10096-024-04862-3)
Supplement: Supplementary file 1 — Supplementary file1 (DOCX 13 KB) [file 10096_2024_4862_MOESM1_ESM.docx]

Supplementary table 1: Criteria for detection of bacteriuria under IFU specifications

|  |  | |  | |
| --- | --- | --- | --- | --- |
| **Criteria as described by IFU** | | **Reference positive** | | **Reference negative** |
| **PA-100 positive** | PA-100 Bacteriuria positive  +  Culture positive for *E. coli, K. pneumoniae, P. mirabilis, S. saprophyticus* and/or *E. faecalis* | | PA-100 Bacteriuria positive  +  Culture negative *for E. coli, K. pneumoniae, P. mirabilis, S. saprophyticus* and/or *E. faecalis* | |
| **PA-100 negative** | PA-100 Bacteriuria negative  +  Culture positive for *E. coli, K. pneumoniae, P. mirabilis, S. saprophyticus* and/or *E. faecalis*  AND  Culture ≥50.000 CFU/mL | | PA-100 Bacteriuria negative  +  Culture negative for *E. coli, K. pneumoniae, P. mirabilis, S. saprophyticus* and/or *E. faecalis*  OR  Culture <50.000 CFU/mL | |
|  | |  | |  |
| **Current routine criteria** | | **Reference positive** | | **Reference negative** |
| **PA-100 positive** | PA-100 Bacteriuria positive  +  Culture positive according to microbiological clinical practice | | PA-100 Bacteriuria positive  +  Culture negative  (incl. contamination or vaginal flora) | |
| **PA-100 negative** | PA-100 Bacteriuria negative  +  Culture positive according to microbiological clinical practice | | PA-100 Bacteriuria negative  +  Culture negative  (incl. contamination or vaginal flora) | |
